# Use, usability, and impact of a card-based conversation tool to support communication about end-of-life preferences in residential elder care – a qualitative study of staff experiences

**DOI:** 10.1186/s12877-022-02915-w

**Published:** 2022-04-02

**Authors:** Therese Johansson, Carol Tishelman, Lars E. Eriksson, Joachim Cohen, Ida Goliath

**Affiliations:** 1 Department of Neurobiology, Care Sciences and Society, Karolinska Institutet, Huddinge, Sweden; 2 Department of Learning, Informatics, Management & Ethics, Karolinska Institutet, Stockholm, Sweden; 3 Stockholm Health Care Services, Region Stockholm, Stockholm, Sweden; 4 School of Health Sciences, City, University of London, London, UK; 5 Medical Unit of Infectious Diseases, Karolinska University Hospital, Huddinge, Sweden; 6 End-of-Life Care Research Group, Vrije Universiteit Brussel and Ghent University, Brussels, Belgium; 7 Stockholm Gerontology Research Center, Stockholm, Sweden

**Keywords:** Palliative care, Advance care planning, Person-centered care, Go wish cards, Patient-provider communication, Qualitative research, Content analysis

## Abstract

**Background:**

Proactive conversations about individual preferences between residents, relatives, and staff can support person-centred, value-concordant end-of-life (EOL) care. Nevertheless, prevalence of such conversations is still low in residential care homes (RCHs), often relating to staff’s perceived lack of skills and confidence. Using tools may help staff to facilitate EOL conversations. While many EOL-specific tools are script-based and focus on identifying and documenting treatment priorities, the DöBra card tool is developed to stimulate reflection and conversation about EOL care values and preferences. In this study, we explore staff’s experiences of use, usability, and perceived impact of the DöBra cards in supporting discussion about EOL care in RCH settings.

**Methods:**

This qualitative study was based on data from two participatory action research processes in which RCH staff tested and evaluated use of DöBra cards in EOL conversations. Data comprise 6 interviews and 8 group meetings with a total of 13 participants from 7 facilities. Qualitative content analysis was performed to identify key concepts in relation to use, usability, and impact of the DöBra cards in RCH practice.

**Results:**

Based on participants’ experiences of using the DöBra cards as an EOL conversation tool in RCHs, we identified three main categories in relation to its usefulness. *Outcomes* of using the cards (1) included the outlining of content of conversations and supporting connection and development of rapport. *Perceived impact* (2) related to enabling openings for future communication and aligning care goals between stakeholders. Use and usability of the cards (3) were influenced by *supporting and limiting factors* on the personal and contextual level.

**Conclusions:**

This study demonstrates how the DöBra cards was found to be useful by staff for facilitating conversations about EOL values, influencing both the content of discussion and interactions between those present. The tool encouraged reflection and interaction, which staff perceived as potentially helpful in building preparedness for future care-decision making. The combination of providing a shared framework and being adaptable in use appeared to be key features for the DöBra cards usability in the RCH setting.

**Supplementary Information:**

The online version contains supplementary material available at 10.1186/s12877-022-02915-w.

## Background

In many developed countries, residential care homes (RCHs) are increasingly major providers of end-of-life (EOL) care for older people [1]. Given the high prevalence of palliative care needs in RCHs [2], it is important to identify appropriate goals of care early in the care trajectory [3]. Proactive conversations about values and preferences for future EOL care (henceforth: *EOL conversations*) may be a way to achieve this, as they have been shown to support value-concordant EOL care provision [4, 5] and reduce decisional conflict and uncertainty among residents’ family members [6]. EOL conversations are integral in *Advance care planning* (ACP), a process that aims to identify, discuss, and document preferences for future care [7]. Yet, considerable variability in ACP delivery and scope of EOL conversations limits strong conclusions regarding its impact on EOL care [8], particularly for older people [9].

Lack of communication about EOL issues in RCHs has been shown to negatively affect perceptions of care quality by e.g., reducing trust in staff’s knowledge about residents’ care preferences [10], whereas repeated open dialogue can allow time for residents and relatives to process information and more carefully consider care goals [11]. However, EOL conversations between staff, residents and/or relatives are infrequent in the RCH context [12], with identified barriers including insufficient skills, experience, and confidence among staff [13, 14]. There is thus a considerable demand for means to support staff in initiating and facilitating EOL conversations in practice. Purely didactic educational programmes, however, have been found to have limited effect. Instead, self-efficacy has been shown to be a stronger predictor for staff engagement in EOL conversations [15, 16], suggesting that competence-building initiatives should include steps to increase practical and emotional capability as well as confidence among staff [17]. One more direct means to strengthen staff’s EOL communication could be through the use of conversation tools.

Existing tools for EOL conversations commonly involve scripts or check-lists that staff follow in discussing and documenting care preferences [18] or are designed as decision aids or for information-sharing, e.g., explaining prognosis and treatment alternatives [19, 20]. These risk supporting unidirectional communication and discussions of medical treatment options rather than stimulating and directing broader reflection about EOL preferences. In contrast, some tools are developed to support both reflection and interactive discussion about values and preferences for EOL care (e.g. Hello [21], Heart to Heart [22], and GoWish cards [23]). In this study, we use the *DöBra cards,* the translated and adapted Swedish version of the U.S. English-language GoWish cards, which cover various physical, practical, existential, and social matters of potential importance for guiding EOL care provision [24]. The English-language GoWish cards have been used to discuss EOL care preferences in various clinical settings [25, 26] and the Swedish-language cards were found acceptable and easy-to-use among community-dwelling older people without known palliative care needs [27, 28]. In addition to clarifying care preferences, the Swedish DöBra cards have been shown to elicit information about underlying values [28] as well as promote interest and confidence in EOL conversations among elder care staff, indicating that the tool has potential to support educational initiatives [29]. Nevertheless, the DöBra cards had not been tested as a tool for care staff to use in EOL conversations with residents and/or relatives in the RCH setting prior to this study.

### Aim

The aim of this study is to explore RCH staff’s experiences of use, usability, and perceived impact of the DöBra cards in EOL conversations with residents and/or relatives.

## Methods

This qualitative study is embedded in a multi-case participatory action research (PAR) project exploring staff competence for EOL conversations in the RCH context, which is in turn part of the national DöBra^1^ research program [30].

The project was conducted in collaboration with Stockholm City Elder Care Bureau, the municipal agency with overarching responsibility for development and follow-up of elder care provision, and the private care company Vardaga. The study thus includes both non-profit and for-profit residential elder care in Sweden. In Sweden there are two forms of residential long-term elder care: RCHs and assisted living facilities (ALFs), which generally cater to residents with fewer medical needs than RCHs. Most of the workforce is comprised of certified nursing assistants (CNAs, also called licensed practical nurses) and nursing assistants as primary caregivers, with at least one registered nurse (RN) always on site and physicians available by phone or at set hours [31, 32]. Since both types of facilities provide round-the-clock access to care staff for daily assistance with e.g., hygiene routines and administration of medication, we refer to them together as RCHs. Neither binding documents nor appointment of proxies are legally admissible in Sweden at present, and ACP is not implemented in Swedish care. There is, however, growing attention on the need for care staff to engage in EOL communication [33, 34] and physician-led “breakpoint” conversations addressing needs and preferences as care shifts from active treatment to comfort care are recommended [35], though insufficiently used in RCHs [36].

### The DöBra card tool

The DöBra cards contain 37 pre-formulated statements about matters of potential importance at the EOL, e.g., “*To say goodbye to important people in my life*”, “*To die at home*” or “*Not being short of breath*” (see [24] for a full list of statements) derived from prior research [37] and ‘wild cards’, which can be used to state any individual priorities not covered by the pre-formulated cards. The cards were translated and adapted to the Swedish context in an iterative process with input from representatives from patient, retiree, health care and community organizations (for details about the development process, see [24]). The procedure for using the cards in this study follows recommedations for the GoWish cards: The cards are sorted in three piles according to their importance to the individual, with the 10 cards chosen as most important cards thereafter ranked. Individuals are encouraged to reflect on and talk about their choices as they sort and rank the cards, providing additional information about underlying reasoning.

### Ethical considerations

The study was approved by the Swedish Ethics Review Authority (ref.no 2017/488–31/4 and 2018/105–32). All participants provided written informed consent and permission to audio-record after having received written and oral information about the study.

### Participants and data collection

This study is based on data generated during two different PAR processes, described below. Characteristics of participating services are provided in Table 1 and an overview of data collection processes is shown in Table 2.

**Table 1:** Characteristics of participating services

	*Service*	*Foci of care*	*Organization*	*Number of beds*
PAR process 1: Staff interviews	RCH A	Somatic care, dementia care	Municipal, non-profit	158
	RCH B	Somatic care, dementia care, psychogeriatric care	Municipal, non-profit	176
	ALF A	Somatic care, social care and activities	Municipal, non-profit	115
PAR process 2: working group	RCH 1	Somatic care, dementia care	Municipal, non-profit	55
	RCH 2	Somatic care, dementia care	Private, for-profit	54
	RCH 3	Somatic care, dementia care	Private, for-profit	75
	ALF 1	Somatic care, social care and activities	Municipal, non-profit	166

*RCH* Residential care home, *ALF* Assisted living facility

**Table 2:** Overview of the data collection processes

	ID	Date	Service(s)	Participants	Duration	Number of EOL conversations held
**Database 1, 2017–2018: Staff interviews (*N*** **= 6)**	I1	Nov 2017	RCH A	2	65 min	None
	I2	Apr 2018	RCH B	2	39 min	1 with resident
	I3	Apr 2018	ALF A	2	58 min	3 with residents, 1 with relative
	I4	Jun 2028	RCH A	2	79 min	1 with relative
	I5	Nov 2018	RCH A	1	94 min	3 with relatives
	I6	Dec 2018	ALF A	2	45 min	None
**Database 2, 2019–2020: Working group meetings (*N*** **= 8)**	WG1	Oct 2019	RCHs 1,2,3, and X^a^	8	107 min	None
	WG2	Oct 2019	RCHs 1,2,3, ALF 1	5	110 min	None
	WG3	Nov 2019	RCHs 1,2,3, ALF 1	7	112 min	10 with residents, 1 relative
	WG4	Dec 2019	RCHs 1,2,3, ALF 1	5	116 min	5 with residents
	WG5	Jan 2020	RCHs 1,2,3, ALF 1	8	106 min	2 with residents, 2 with relatives
	WG6	Feb 2020	RCHs 1,2,3, ALF 1	4	111 min	2 with relatives
	WG7	Oct 2020	RCHs 1,2, ALF 1	3	102 min	Not possible due to Covid-19
	WG8	Dec 2020	RCHs 1,2, ALF 1	4	86 min	Not possible due to Covid-19

*RCH* Residential care home, *ALF* Assisted living facility

^a^X = RCH that withdrew following the first meeting

#### Database 1. Interviews about experiences from staff-led initiatives with the DöBra cards

The first database derives from interviews conducted in 2017–2018 with elder care staff who notified us about staff-led initiatives involving the DöBra cards in three services following participation in a previous study (for study details see [29]). To learn about the experiences of using the cards in the RCH setting, follow-up conversational interviews (*N* = 6) were conducted with five participants (Table 2). Interviews were audio-recorded and professionally transcribed verbatim. Authors TJ (doctoral student, MSc. in psychology) and IG (PhD and RN with an extensive background in EOL care practice and research) conducted the interviews together, with one exception, conducted by TJ alone. Both interviewers were known to the participants from the previous study [29].

#### Database 2. Meetings with a working group for co-developing practical guidance for EOL conversations using the DöBra cards

This database derives from meetings over time with one working group, comprising researchers and elder care staff, established in 2019. The goal was to explore how the DöBra cards might be used in EOL conversations with residents and/or relatives and co-develop written guidance based on lessons learned. The recruitment process was two-phased. First, convenience sampling was used to invite services through existing collaborations with Stockholm City Elder Care Bureau and Vardaga. Second, a contact person in each service recruited one RN and one CNA to join the working group. Initially, four RCHs and one ALF expressed interest in participating. However, one RCH withdrew after the first meeting, stating lack of time to fully engage. The working group thus comprised eight recurring participants from four services who tested and evaluated use of the cards in EOL conversations, discussing their experiences in regular meetings (*N* = 8) that were audio-recorded and professionally transcribed verbatim. Using topic guides informed by the cyclical PAR process of planning, action, reflection, and evaluation [38], the meetings were facilitated by authors TJ and IG, unknown to the working group participants prior to the study. Hosting rotated, with meetings either held in conference rooms at a university or one of the participating services. The two final meetings were conducted online using the video-conference platform Zoom, due to the Covid-19 pandemic. The online meetings followed the same procedure as the physical meetings and were audio-recorded using an external device only accessible to the researchers so that no data were collected or stored on the video-conference platform or related cloud services. An overview is shown in Table 2, with details about aims and procedures for the co-development process provided in Supplement file 1. Data analysed here include meeting transcripts and minutes.

### Data analysis

Inductive qualitative content analysis (QCA) was conducted to explore staff experiences of using the cards. Following each interview/meeting, author TJ wrote reflective notes which served to initiate the analytic process concurrently with ongoing data collection. Formal analysis was initiated and led by TJ who first read all transcripts to become familiar with the data. Following Kyngäs’ approach to inductive QCA [39], the analytic process followed stages of data reduction, data organization, and data abstraction. Open codes related to the research aim were constructed in NVivo. Pro (version 11) based on the meaning of the manifest content [39]. These codes were indexed and grouped based on similarity in content to form categories in an initial coding scheme that was iteratively developed and revised throughout the analytic process. To ensure analytic rigour, the coding scheme and preliminary findings were critically reviewed in discussion with all authors to discuss alternative interpretations [40]. Supplement file 2 details the full coding scheme, exemplified with the quotes used below and additional quotes. After completed analysis, we noted the relevance of a Theory of Change (ToC) [41] approach for conceptualizing our findings. ToC is a framework to describe how an intervention moves from input to impact, by a “backwards mapping” of intermediate outcomes acting as preconditions for long-term outcomes and the influence of contextual factors [42], which inspired the presentation of the results.

## Results

In total, data deriving from 11 women and 2 male staff members, aged 32–65 years, working in seven elder care services were analysed in this study. Seven were CNAs, five RNs, and one was an activity coordinator, and they had worked in elder care between 1 and 40 years (median = 18 years). Additional demographic characteristics of the participants are shown in Table 3. In the working group, attendance varied from three to eight participants/meeting (median = 5), primarily due to lack of replacement staff and sick leave.

**Table 3:** Demographic characteristics of participants in both data collection processes

Participants	*N* = 13
**Gender**	
Women	11
Men	2
**Profession**	
Certified nursing assistant (CNA)	7
Registered nurse (RN)	5
Activity coordinator	1
**Education (highest qualification)**	
Upper secondary education	7
Higher vocational education diploma	1
University diploma (> 3 yrs)	5
**Place of birth**	
Sweden	7
Europe excl. Sweden	3
Africa	1
Asia	1
South America	1

**Fig. 1:**
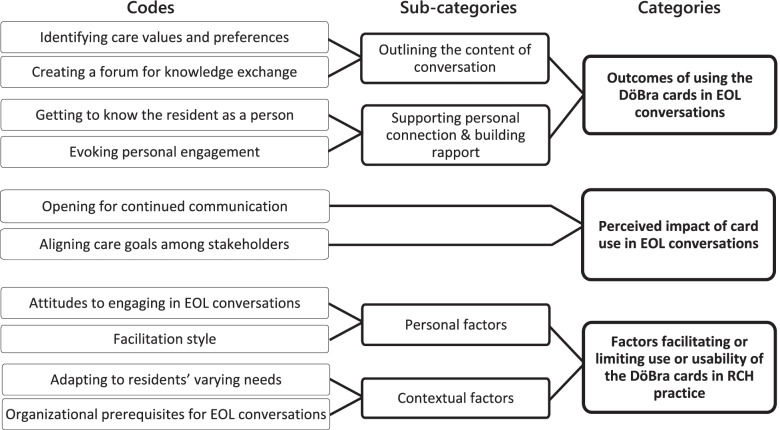
Code tree showing the codes, sub-categories, and categories

**Fig. 2:**
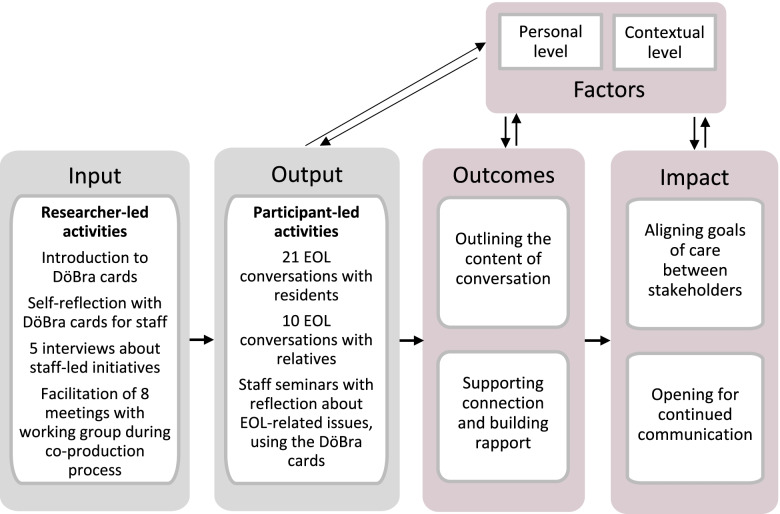
Schematic Theory of Change model showing project process and findings. Note: Inspired by the Theory of Change (ToC) framework, we illustrate the results in relation to study input, i.e., the activities performed by researchers, and output, i.e., activities performed by participants. Arrows illustrate the influence of personal and contextual factors on output, outcomes, and perceived impact of using the DöBra cards in EOL conversations in RCH practice

Figure 1 shows the main categories, sub-categories, and codes constructed through data analysis. Figure 2 illustrates the results in chronology and presents inter-relationships between input, output, and the main categories of the findings. Analytic points are illustrated with quotes, linked to profession and data collection stage, i.e. interview or working group meeting number, to demonstrate the link between data and findings [43] while maintaining individual partcipants’ anonymity.

### Category 1: outcomes of using the DöBra cards to discuss EOL values and preferences

Participants’ experiences of using the cards in EOL conversations with residents and/or relatives highlighted two main outcomes of using the DöBra cards: they outlined the conversation by providing a shared vocabulary and a framework, and supported the development of personal connection and rapport through an intimate discussion of EOL-related values (Figs. 1 and 2).

#### Outlining the content of the conversation

The cards were said to provide a conversational structure, with the preformulated card statements serving as concrete examples triggering reflection and discussion. In this manner, the tool was useful for exploring care preferences in general and in detail, promoting personal stories and previous EOL experiences to be shared, and enabling residents and/or relatives to ask questions about EOL care provision.

##### Identifying care values and preferences

A central contribution of using the cards was said to relate to their support in eliciting and clarifying information about personal values that could guide person-centred care both at present and later, at the EOL. Card statements were described as concrete and straightforward, helping participants initiate EOL conversations by addressing specific matters. Residents and relatives were said to have something to react to, creating a shared point of departure for conversation. The cards were seen as stimulating reflection about EOL values and preferences and enabling these to be communicated, as suggested by this RN: *“There were some statements that were chosen that you didn’t know about her.* … *So, some things became much clearer*” (WG2). Although card statements were described as useful, some staff said the statements were too generic on their own, with conversations about them necessary to provide information specific enough to guide person-centred EOL care.

##### Creating a forum for knowledge exchange

The cards seemed to help make EOL conversations reciprocal learning opportunities, in which knowledge about the resident as a person and information about care routines were discussed. This is exemplified as participants in the working group discuss what EOL conversations entail:

CNA1: *No, it’s a conversation, actually. It’s mutual and...*

CNA2: [interrupting] *Knowledge exchange would be more appropriate perhaps.*

RN: *… it’s easier, in some ways, to just conduct an interview with the cards* (WG6)

Use of the cards was also described as allowing staff to explain how they could assist at the EOL. For example, one RN related how, during the card sorting, a resident was able to pinpoint a specific concern, leading to a constructive discussion about how this might be alleviated: *“*[The resident] *said ‘I’m afraid of being scared‘.* … [I said] *‘in what way do you think we could help?’ So we talked about that now there are medications … that can help with anxiety and so on”* (WG3). A CNA referred to a conversation in which a relative, in response to a specific card statement, started asking questions that made the participant realize how little some relatives may know about EOL care:” [the relative] *asked me a very strange question. ‘When my mother dies, what will happen to her?’* … *For us, it’s so obvious, but for a relative, she didn’t know”* (WG5). This highlights staff’s increasing awareness of the imbalance between their knowledge about EOL care and that of residents or relatives, and recognition that questions about EOL care may not be posed without opportunity to consider such issues.

Card statements also triggered sharing of personal stories, which were said to be helpful in elucidating EOL care preferences and the underlying reasoning for them. One CNA reflected on this after a conversation with a resident with a history of mental illness who had previously spoken of ending her life:

*Everyone thought she only lives in her own world, no one can trust her 100%.* … *But during the conversation, I thought, my God, she’s a completely different person, why have we had preconceived notions about her instead of talking to her and understanding what kind of person we have in front of us.* (WG4)

The CNA spoke of learning that the resident did not feel like she was finished living yet, triggered by the card ‘to have lived my life to the fullest’. Sharing personal stories was not only said to be positive, however, as personal stories were sometimes described as “taking over” the perceived purpose of the conversation. For example, the same CNA spoke of how another resident kept coming back to his guilt about the past when sorting through the DöBra cards:

*it was very difficult to complete the conversation* … . *He felt guilty because* … *he didn’t have enough time with his* [deceased] *wife and so on.* … [The conversation] *became completely different because he only said three things that were important to him.* (WG4)

Thus, sharing of EOL-related stories could provide rich information about values and preferences but might also make it more difficult for staff to direct the EOL conversations in the manner they had expected. However, the working group discussed this, suggesting using active facilitation by asking questions about other cards to lead back to discussion of EOL values.

#### Supporting personal connection and building rapport

As noted above, conversations about the EOL could trigger memories and existential thoughts, and participants found that some EOL conversations thus created an intimate atmosphere and a sense of rapport. In addition, the DöBra cards seemed to support a shared moment of reflection and insight that participants suggested contributed to forming a closer connection with the resident, which increased their investment in future care provision.

##### Getting to know the resident as a person

By promoting reflection and intimate conversation about various aspects of the EOL, the cards were said to elicit a more comprehensive understanding of the resident. Participants described how they got to know the individual on a more personal level: *“I thought ‘how great, now I can say that I really know my resident’.* … [Her chosen cards reflected] *her personality, for example she still always has lipstick on”* (RN, WG3). EOL conversations also seemed to constitute memorable interactions in an emotionally salient situation, as suggested by another RN: “*I almost got the feeling that it became... something magical that you cannot put your finger on when you sit there. That you get very close to each other, like chemistry”* (WG3). This closeness was discussed by the participants as a foundation for building trusting relationships with residents and/or relatives, with staff hoping that this could help make future EOL conversations easier.

Even in EOL conversations that were conducted with relatives alone, participants described getting a better sense of who the resident was as a person through detailed stories about the resident’s life. However, participants pointed out that it could become difficult to differentiate relatives’ understanding of residents’ EOL values and preferences from relatives’ own preferences: “*That became difficult for [the relative] to keep apart, I think, what was important to him and what was important to his wife”* (RN, WG6).

##### Evoking personal engagement

Participants spoke of how engaging in the conversations with residents and/or relatives supported mutual trust, engagement, and assurance. One RN exemplifies this, reflecting: *“after you have used the cards … it feels like if I would provide palliative care for* [the resident]*, she’d have more confidence in me now”* (WG2). At a later meeting, the same RN referred to this conversation again, suggesting it had left a lasting impression: “*during my parental leave she passed away, and I’ve thought about what a nice meeting we had before and that I got a little closer to her then”* (WG7). Participants noted that the procedure of sorting and ranking the cards sometimes led to focusing overly on the tool, limiting dynamic conversation and connection. While it was acknowledged that these experiences may still be valuable and allow individuals to reflect about rarely addressed matters, it became difficult to actively facilitate EOL conversations when the resident or relative *“just … sorts cards and almost doesn’t talk for half an hour”* (I5).

Similarly, in EOL conversations with more than one person, residents and relatives would sometimes talk amongst themselves. Such situations were said to sometimes make staff members uncertain about how to act, e.g., whether they should try to engage or simply observe and listen. One RN talked about an EOL conversation with a resident and her daughter during which the daughter read the card statements aloud for her mother and sorted them for her: “*Maybe one should have played a larger role in some way* … *Well, you feel* … *less involved when there’s another person present and they’re talking a lot”* (WG5).

This illustrates how less active participation may decrease participants’ sense of closeness and connection with the resident and/or relative. At the same time, participants noted that such situations may contribute to closeness between those present, which was also recognized as beneficial.

### Category 2: perceived impact of card use in EOL conversations

In addition to the outcomes of card use that supported the process of EOL conversations, participants perceived more lasting impacts of card uses in EOL conversations (see Figs. 1 and 2). Such impact affected the individuals involved in the discussion and relationships between them.

#### Opening for continued communication

Participants generally acknowledged that EOL conversations were not one-off events but a starting point for continued reflection and discussion, which could be revisited and gradually developed further, as suggested by this CNA:

*I think this isn’t just a meeting,* [rather] *a process that must be complemented mutually. Sometimes you* [start] *but you don’t have time* … *and you have to end the conversation … and say “Okay, now we have started talking about it and I will return.”* (WG2)

The need for iterative conversations was further emphasized by participants’ recognition of EOL preferences as dynamic and changing over time. By using the cards, participants highlighted that relatives could become more aware of the residents’ values and preferences and residents could identify values that they might not previously have spoken about. Participants also noted how increased future communication about EOL matters between residents and relatives without staff facilitation could potentially build preparedness for future decision-making.

#### Aligning goals of care among stakeholders

EOL conversations using the DöBra cards were also a means to enable staff, residents, and relatives to clarify and align future goals of care at the EOL. One RN spoke about an EOL conversation with a relative who repeatedly had expressed dissatisfaction with care at the RCH. Using the cards had provided opportunity for a discussion about what the EOL might be like for a patient with Alzheimer’s disease and allowed the participant to explain how staff could work to fulfil care priorities. Soon after that conversation, the resident’s health deteriorated further and the relative visited more often:

*RN: I felt that somewhere he had gained more understanding. I think it was thanks to the cards because he no longer talked about needing to send her somewhere else, not even when I raised the issue.* …

*Interviewer: How was the conversation with him?*

*RN:* … [while using the cards] *he started to think out loud … that if he himself would become demented, one would hardly want anything unnecessary but would want to have peace and quiet and have things be as good as possible.* … [So] *we were in full agreement in this final period, that we should try to make it calm* [for her]*.* … *And I connected it to the cards, that we had had this shared moment.* … *He said he had not thought of such things at all before.* (I5)

Here, the EOL conversation became the first step in connecting with a relative described as previously being confrontative with staff. The cards appeared to become a tool for creating common ground between the relative and the RN in terms of the resident’s care, making it possible to agree on care provision at the EOL.

### Category 3: factors that influence use and usability of the DöBra cards

Several factors on the personal and contextual level facilitated or limited use of the cards and their perceived usability in this context. These factors relate to staff’s personal traits and attitudes, and contextual features of both the conversation and the RCH organization (Fig. 1).

#### Personal factors

Participants’ personal traits primarily influenced cards use directly, as they affected their willingness to use the cards.

##### Attitudes to engaging in EOL conversations

From the outset, participants’ attitudes towards EOL conversations influenced their motivation to use the DöBra cards in practice (Fig. 2). Those who originally spoke of the usefulness of EOL conversations in RCHs were more positive to the tool, whereas participants who seemed more sceptical about the value of EOL conversations also expressed more hesitation about testing the cards. Several participants were initially uncertain about the tool, saying the cards addressed EOL issues too directly. Over time reluctance seemed to dissipate, as one RN said: *“almost all of us were also rather sceptical before, if you think about the first meeting, and now we are sitting here and are quite positive”* (WG5). Hence, experience of discussing EOL issues and self-perceived competence influenced participants’ use of the tool. Participants with limited previous experience of EOL conversations and those describing death as difficult to discuss said the cards could act as a catalyst. In contrast, participants describing themselves as experienced often had their own ways of broaching the EOL. For them, the cards were occasionally said to hinder “natural” conversations, and the format and imposed structure could be described as constraining.

Another factor that affected participants differentially was the name, the DöBra cards, as it is a pun in Swedish that means both “*dying well*” and *“awesome”*. Though some participants appreciated that the word death was explicit, many found the name blunt and provocative. Furthermore, the Swedish word for a deck of cards (*kortlek*) literally means *“card game”*, said to further imply that EOL conversations involved playing: “[it] *makes you take it less seriously and* [people] *may not understand the purpose because they just hear ‘game’, it becomes silly*” (RN, WG3). While participants mentioned how some residents and relatives commented the name, participants’ concern about risk being provocative generally eased over time.

##### Facilitation style

Participants’ descriptions suggested varied facilitation styles, which in turn seemed to affect direction and depth of EOL discussions. Curiosity about the resident or relative was described as imperative as was being comfortable with emotional reactions; apprehension about triggering negative emotional reactions or damaging trust between staff and residents/relatives seemed to hinder participants’ willingness to ask follow-up questions or conduct EOL conversations at all.

Participants’ ability to be flexible and adaptable to different needs and circumstances was identified as a major requirement for the cards to be useful in the RCH setting. Being able to give up control of the conversation and respond to the resident’s/relative’s reactions appeared essential:

*I understood that for her it wasn’t at all important to use the cards. She simply wanted to talk* … *And I listen.* … *Mostly affirming* [what was said]*.* … *No, you don’t talk much. you mainly bring up the questions.* (CNA, WG6)

This highlights EOL conversations as having variety of potentially valuable end-results, depending on the flexibility of the participant in facilitating a shared endeavour conducive for engaged conversations and rapport.

#### Contextual factors

Contextual factors, beyond individual staff member’s control, affected both their possibility to conduct EOL conversations in practice as well as their perceptions of card usability.

##### Adapting to residents’ varying needs

The variation in cognitive and physical status among RCH residents made it clear that the default card procedure was not always feasible or appropriate. The 37 card statements, with perceived overlap between some, were occasionally said to make the process of sorting and prioritizing cards both time-consuming and cognitively demanding. EOL conversations with residents with cognitive decline, impaired sight, or loss of motor skills could therefore require the card exercise to be adapted, e.g., participants sometimes read the card statements aloud or turned them into questions. Participants appeared mindful of adjusting the conversation to the individual, as highlighted by one CNA: *“We should help people, we shouldn’t expose them to stress and anxiety and failure*” (WG6). Sometimes only minor alterations were required when participants noticed that a resident was becoming tired, restless, or struggling to choose cards. For example, participants described skipping the ranking component or letting the resident/relative choose important cards without sorting them. Generally, participants argued that it was not suitable to use the cards with residents with severe dementia, perceiving greater risk of harm and lower potential gain due to the complexity of the card exercise. Instead, participants suggested that the cards could be used with relatives of such residents.

##### Organizational prerequisites for EOL conversations

Since EOL conversations could be emotional, participants emphasized the need for a calm, quiet, and secluded environment, preferably residents’ own apartments though meetings rooms or empty activity rooms were also said to be appropriate. Being able to allot uninterrupted time was also identified as vital for maintaining presence and attentiveness: “[staff] *can’t do anything besides be involved in the conversation* [during the allotted time]... *Because you absolutely don’t want to be interrupted* … *so one needs to be completely spared* [from other duties] *for like an hour”* (RN, WG6). Interruptions or having to hurry to complete the card exercise were argued to be detrimental, as both the discussion and the atmosphere would be affected. Use of disparate documentation systems between services and between staff categories within the same service hindered written communication about residents’ EOL values and preferences to be shared effectively, and thus also affected usability.

## Discussion

Based on exploratory qualitative analysis of data from both non-profit and for-profit RCH and ALF services about staff experiences of using the DöBra cards to discuss future EOL care, we identify the perceived outcomes, impact, and usability of a novel EOL conversation tool. This is particularly relevant in contexts, such as Swedish elder care, where such discussions are infrequent. We found that the cards can be directly useful, by providing a framework to structure EOL conversations, helping to elicit valuable information about residents’ EOL values and preferences, and enabling an interpersonal connection that strengthens rapport. In addition, our findings suggest that the cards may have more long-term impact by encouraging continued communication among stakeholders, with or without staff facilitation, and aligning care goals by supporting discussions to clarify what matters at the EOL. Factors that influenced use and usability related to both characteristics of staff members and contextual features, based on the needs and health status of the resident involved, as well as organizational features.

Given the novelty of facilitating proactive EOL conversations in elder care, and using a conversation tool to do so, a qualitative study design was crucial to comprehensively explore staffs’ experiences, and reflections regarding use and usability. Our prolonged engagement with participants may have contributed to a sense of trust for participants to openly share their insights and experiences and engagement in the study was high, suggesting that the study focus was perceived as relevant. Furthermore, using a participatory approach during data collection enabled preliminary findings to be discussed with participants throughout. This was particularly the case in the working group in Database 2, where sharing and discussing data and initial interpretations was part of the process of co-developing the written guidance. While member-checking does not verify results, general agreement and recognition of findings strengthens their trustworthiness [44]. Nevertheless, it should be remembered that these data reflect only participating staff’s views of the EOL conversations and subsequent occurrences. It is worth noting that only staff who contacted the authors after having used the DöBra cards were interviewed for Database 1. It is possible that other staff utilized the tool without notifying us. Staff with more negative experiences might have been less likely to make contact, leading to under-representation of such experiences. Likewise, in Database 2, it is possible that participants who remained in the working group were more positive to testing an EOL conversation tool than others. Thus, self-selection may constitute a potential bias that should be remembered when considering transferability of the findings.

Unlike other, often script-based, EOL conversation tools, the cards provided examples that could be freely explored, serving as a framework stimulating in-depth reflection about EOL values and preferences. Our study adds more detailed understanding about how using the DöBra cards as a tool in EOL conversations can affect several important aspects of interaction. In addition to helping staff address and ask about residents’ EOL values and preferences, the cards stimulated a reflective process and provided interactive sharing of information, stories, and emotions. These findings highlight the interpersonal aspects of EOL conversations, e.g., strengthening relationships and developing shared narratives and goals, which have been shown to act as major contributors to ACP benefit [45], particularly with older populations [46, 47]. We found, as did Sussman et al. [48], that interactive tools, such as card games, help target reflection and can cover a variety of aspects of EOL care. However, since most EOL conversation tools focus primarily on medical aspects of care, other important dimensions risk being overlooked [48]. Prior research has suggested that non-medical issues may even be more imperative to discuss as advanced age affects care preferences [49]. As the DöBra cards cover physical, practical, existential, and social matters, they offer a more comprehensive perspective on EOL values and preferences beyond medical treatment options alone.

The DöBra cards were found to be useful to strengthen person-centered care provision and rapport both during and after EOL conversations. This is in contrast to a recent study by Groebe et al. [50], in which some care staff considered EOL conversation tools to be counterintuitive to an individualized care approach. It may be that the physical format of the DöBra cards better allowed residents and/or relatives to be actively involved in directing the discussion than other conversation tools, as the cards they choose served as route markers for mapping the discussion, with as much – or little – commentary as residents and/or relatives wished. However, this required considerable flexibility and attentiveness from staff in facilitation. Our findings thus highlight the delicate balance between seeing the cards as a tool to complete a task with a set goal on the one hand or as a trigger for an unfolding conversation in which involved parties together determine issues important to discuss on the other. This raises questions about how underlying goals of EOL conversations should be negotiated and determined. Using the DöBra cards might create forums for sharing stories or thoughts that residents and/or relatives need to express and allow discussion of topics that otherwise would not be addressed, making them meaningful experiences, even if they do not directly contribute to, or even risk hindering, completion of the card exercise as planned. We propose that staff instructions for card use need to be carefully considered as to not create perceptions of failure if the card exercise cannot be completed, which is important to consider when designing competence-building initiatives and in future research on tools for EOL conversations.

The results of this study also strongly suggest that there is no optimal ‘one size fits all’ procedure for using the cards and that each EOL conversation needs to be adjusted to fit the needs and constraints those participating, in line with suggestions in prior research [51, 52]. The flexibility of DöBra card use therefore appears to be a key feature for their potential usability in RCHs and is particularly important if the tool is to be used with residents with cognitive decline. This is an important finding since previous research with the DöBra cards has primarily focused on relatively well-functioning community-dwelling older people [27, 28]. In general, EOL conversations are rarely conducted with residents with dementia [53] and participants’ experiences highlighted cognitive function as a challenge, even though this study primarily involved residents who staff considered cognitively competent. The expected increase in dementia prevalence further emphasizes the need to find feasible ways to support EOL conversations also with this population [54, 55]. In these cases, while the ranking exercise appeared too complex, the card statements were still useful as probes to clarify what matters at the EOL, as also noted by Eneslätt et al. [27]. Nevertheless, the usefulness of the DöBra cards to discuss EOL values and preferences with residents with moderate and severe cognitive impairment needs to be studied further [56]. In addition, future research should include residents’ and relatives’ perspectives of using the DöBra cards.

The cultural and linguistic diversity among participants adds to the external validity of this study, reflecting the multi-ethnic workforce in Swedish elder care [57]. Cultural and religious beliefs and practices are known to shape care preferences as well as communication styles [58] and language barriers between stakeholders can hinder EOL communication [59]. Our data, however, suggest that neither use nor usability of the DöBra cards was influenced by cultural or linguistic factors, but rather differences in attitudes to EOL communication. Additional research is required to better understand if, and how, using the DöBra cards might bridge linguistic or cultural barriers to EOL conversations.

Our results show that staff found it easier to discuss EOL issues using the DöBra cards, but also add to the extant research that indicates that communication skills are a precondition for EOL conversations [60, 61]. The direction and depth of EOL conversations seemed largely influenced by staff’s interest and curiosity about what matters to other people. Additionally, the practice of ‘holding space’, i.e. actively listening, being mentally and emotionally present, and setting aside one’s own agenda to allow the other person to lead [62] appeared to constitute a key skill for facilitating EOL conversations described as richer and more memorable. This highlights that EOL conversations differ from other conversations, by requiring staff to respond to existential needs and shift their focus from ‘doing’ towards ‘being’ and listening [63]. Building such skills may require a more experience-based approach to training, as suggested by Sand et al. [64].

The influence of contextual factors on implementation of EOL conversations is well known and this study corroborates several previously identified barriers, such as unclear mandates, under-staffing, and negative staff attitudes [65–67], as well as new prerequisites, e.g., related to systems for shared written documentation. In addition, we extend understanding about how time constraints influence not only prevalence of EOL conversations, but also their depth and salience, as stress when EOL conversations took longer than expected was a source of frustration or impatience among participants. These findings point to fundamental challenges in introducing new processes in care systems already pressed for time and resources, and as argued by Lund et al. [60], it may be that until these are dealt with, the benefits of using tools to support EOL conversations will be limited. Impact of wide-scale implementation of EOL conversations using the cards into routine RCH practice thus remains a critical question for future research to explore.

## Conclusion

Even though proactive conversations between care recipients, their relatives and care providers to discuss values and preferences for future care have been pointed out as central for person-centered care provision at the EOL, such communication is still not widely adopted in residential elder care. This study contributes with valuable knowledge as RCH settings are increasingly becoming major providers of EOL care, as we demonstrate ways in which a novel tool, the DöBra cards, were found useful by staff to facilitate discussions about EOL values and preferences with residents and/or relatives. Direct outcomes of using the tool related both to content, i.e., elicitation of valuable information to guide future care, and interaction, i.e., development of interpersonal connection and rapport. In the long term, conversations with the DöBra cards may help prepare stakeholders for future EOL care decision-making by enabling communication and increase consensus around care goals. Specifically, this study highlights two features that appear to strengthen mutual interaction and knowledge exchange about EOL matters: a physical conversation tool format, which served as a shared structure for the discussion; and the flexibility in ways to use the DöBra cards to fit individual needs, which contributed to the helpfulness of the tool even with residents with mild cognitive decline. This combination of structure and adaptability in EOL conversations enabled residents and relatives to, together, actively direct the course and depth of the conversation using the DöBra cards.

## Supplementary Information

**Additional file 1.**

**Additional file 2.**

## Data Availability

The datasets used and/or analysed during the current study are available from the corresponding author on reasonable request.

## Abbreviations

ACP: Advance care planning, the formalized process of discussing and documenting preferences for future end-of-life care
ALF: Assisted living facility
CNA: Certified nursing assistant, also known as licensed practical nurse (LPN)
EOL: End-of-life
RCH: Residential care home
RN: Registered nurse
QCA: Qualitative content analysis
ToC: Theory of change

## Footnotes

1 “DöBra” is a Swedish pun that literally means dying well, but figuratively means awesome
