# Supplementary material for: Use, usability, and impact of a card-based conversation tool to support communication about end-of-life preferences in residential elder care – a qualitative study of staff experiences
Source: BMC Geriatr. 2022 Apr 2;22:274. doi: 10.1186/s12877-022-02915-w (PMC8976536; doi:10.1186/s12877-022-02915-w)
Supplement: Supplementary file 1 — Additional file 1. [file 12877_2022_2915_MOESM1_ESM.docx]

*Supplement file 1*. Overview of the process for co-producing the practical guidance with the working group in Database 2

| **Meeting** | **Aim and procedure** | **Assignment* for next meeting** |
| --- | --- | --- |
| 1 | Introduce the DöBra cards to the participants (each is given one card deck);  Explore participants’ experiences of EOL conversations;  Describe the purpose of the working group, i.e. to construct practical guidance on using the DöBra cards in elder care based on lessons learned from small-scale testing and evaluation;  Set ground rules for the group.  Procedure: group discussion, self-reflection using the DöBra cards | Use the DöBra cards with a friend, colleague, family member, etc.  Learning goals: practice a) introducing the DöBra cards and explain what they are used for, and b) practice facilitating someone else in using the tool to reflect on and discuss EOL values and preferences |
| 2 | Discuss experiences of using the DöBra cards in EOL conversations by asking questions such as: What happened during the conversation? What worked well and what was challenging? What did you learn about the other person?  Identify ways to inform others at the service about the project focusing on what should be communicated, who should be informed, and how the information should be shared.  Procedure: group discussion, brainstorming exercises using post-its | Invite residents and/or relatives to EOL conversations. Facilitate at least one EOL conversation with a resident or relative using the DöBra cards (following original instructions).  Learning goals: Practice informing others about EOL conversations. Test the DöBra cards’ original instructions in EOL conversations with target audience. |
| 3 | Explore strategies for inviting residents/relatives to EOL conversations, e.g. identify ”key phrases” that can be used;  Explore experiences of using the DöBra cards in EOL conversations (probing questions exemplified above);  Given the need for written information identified in meeting 2: Discuss what should be included in an information brochure about EOL conversations.  Procedure: group discussion, brainstorming exercises using post-its | Facilitate an EOL conversation with a resident or relative (adapt DöBra card procedure if necessary).  Learning goals: Practice facilitating EOL conversations using the DöBra cards. Reflect on how one initiates and ends EOL conversations. |
| 4 | Explore experiences of using the DöBra cards in EOL conversations (probing questions exemplified above);  Discuss a prototype for an information brochure about EOL conversations, aimed towards residents and/or relatives.  Procedure: group discussion | Facilitate an EOL conversation with a resident or relative (adapt DöBra card procedure if necessary). Document information generated in an existing record system.  Learning goals: Practice facilitating EOL conversations using the DöBra cards. Reflect on what information should be documented and where it is most appropriate to do so. |
| 5 | Explore experiences of using the DöBra cards in EOL conversations (probing questions exemplified above), focusing on particular features of facilitating a joint EOL conversation;  Identify what information should be documented and the alternative record systems available for such documentation.  Identify situations in which use of the DöBra cards is challenging and discuss alternative procedures to handle these;  Procedure: group discussion, brainstorming exercises using post-its | Facilitate an EOL conversation with a relative or a resident and relative together (adapt DöBra card procedure if necessary). Document information generated. Reflect on prerequisites for more wide-spread implementation of EOL conversations with the DöBra cards in the service.  Learning goals: Practice conducting EOL conversations using the DöBra cards. Identify organizational needs for continued uptake and implementation of EOL conversations. |
| 6 | Explore experiences of using the DöBra cards in EOL conversations (probing questions exemplified above);  Discuss revised prototype for an information brochure about EOL conversations, aimed towards residents and/or relatives;  Reflect on one’s own role in the EOL conversation, identifying  Procedure: group discussion, brainstorming exercises using post-its | Facilitate an EOL conversation with a resident and/or relative using the DöBra cards (adapt original instructions if needed). Document information generated. Teach a colleague to facilitate an EOL conversation with the DöBra cards.  Learning goals: Practice facilitating EOL conversations using the DöBra cards. Identify organizational needs for continued uptake and implementation of EOL conversations. |
| 7 | Explore the impact of the COVID-19 pandemic in the participants’ services and how it has affected the project;  Review a first draft for the practical guidance (based on group discussions, written by TJ and IG)  Procedure: online group discussion | None given, but participants were encouraged to continue with EOL conversations if possible, given the circumstances, and to document the information generated. |
| 8 | Explore the continued impact of the COVID-19 pandemic in the participants’ services and how it has affected the project;  Discuss a revised draft for the practical guidance (written by TJ and IG)  Procedure: online group discussion | None given, but participants were encouraged to continue with EOL conversations if possible, given the circumstances, and to document the information generated. |

Notes: EOL= end-of-life

*All assignments were voluntary
