# Supplementary material for: Use, usability, and impact of a card-based conversation tool to support communication about end-of-life preferences in residential elder care – a qualitative study of staff experiences
Source: BMC Geriatr. 2022 Apr 2;22:274. doi: 10.1186/s12877-022-02915-w (PMC8976536; doi:10.1186/s12877-022-02915-w)
Supplement: Supplementary file 2 — Additional file 2. [file 12877_2022_2915_MOESM2_ESM.docx]

*Supplement file 2.* Code matrix showing the final coding scheme with examples of coded data. The matrix contains the quotes used in the article text along with additional quotes to illustrate the data in more detail. Sources of quotes are presented with reference to data collection stage, e.g. interview (I 1-6) or working group meeting (WG 1-8). Professions, occasionally numbered, are presented in cases of interactions between participants to increase clarity, but do not denote any individual person to protect the anonymity of participants.

| **Examples of data** | **Codes** | **Sub-categories** | **Category** |
| --- | --- | --- | --- |
| *It became a bit more structured, in my experience* … *and it felt easier to be able to start somewhere.* (working group meeting 1 (WG1))  *There were some statements that were chosen that you didn’t know about her* … *So, some things became much clearer.* (WG2)  CNA1*: She would like to have* [someone] *with her to hold her hand the day when it's time*  RN1*:* [She wants to] *die at home?*  CNA1*: Yes, do not send* *in* [to hospital]*. She wants to be home* [at the RCH] *when she dies, and she wants loved ones around her at that time.*  RN1*: That is very important information*. (WG3)  [The] *woman I interviewed last time, she chose* [the card] *‘to be cared for by staff that knows me well’. And it was interesting … when she would choose* [the top] *10 cards, she chose the staff* [card and not to have her family present]. *I asked, why did you choose the staff? ‘Well, I'm already done with* [my] *relatives.* … *I want a person who will know me.’* (WG4) | Identifying care values and preferences | **Outlining the content of conversation** | **Outcomes of using the DöBra cards in EOL conversations** |
| CNA1: *No, it’s a conversation, actually. It’s mutual and...*  CNA2: [interrupting] *Knowledge exchange would be more appropriate perhaps.*  RN: *… it’s easier, in some ways, to just conduct an interview with the cards* (WG6)  [The resident] *said ’I'm afraid of being scared‘.* … [I said] *‘in what way do you think we could help?’ So we talked about that now there are medications … that can help with anxiety and so on.* (WG3)  *Many questions were raised as well, medical questions and explanations. They needed to know, and I explained.* (WG4)  *And* [the relative] *asked me a very strange question. ’When my mother dies, what will happen to her?’* … *For us, it’s so obvious, but for a relative, she didn’t know.* (WG5).  *Everyone thought she only lives in her own world, no one can trust her 100%. … But during the conversation, I thought, my God, she’s a completely different person, why have we had preconceived notions about her instead of talking to her and understanding what kind of person we have in front of us.* (WG4)  *It was very difficult to complete the conversation* … *He felt guilty because* … *he didn’t have enough time with his* [deceased] *wife and so on.* … [The conversation] *became completely different because he only said three things that were important to him.* (WG4) | Creating a forum for knowledge exchange |  |  |
| *I thought ‘how great, now I can say that I really know my resident’. …* [Her chosen cards reflected] *her personality, for example she still always has lipstick on.* *That's the first thing she does* [in the morning] *... And she chose as an important* [card]*, that I want to be clean and neat (laughs).* (WG3)  *I almost got the feeling that it became... something magical that you cannot put your finger on when you sit there. That you get very close to each other, like chemistry*. (WG3)  *I felt that she enjoyed talking* [about the resident]*. She thought it was a good time to sit and talk about* [him]*. Mostly what he has done in his life. It became like a life story, I thought.* (WG6)  “*That became difficult for* [the relative] *to keep apart, I think, what was important to him and what was important to his wife*” (WG6). | Getting to know the resident as a person | **Supporting personal connection & building rapport** |  |
| *… after you have used the cards, there is… a feeling that you got closer to each other. And it feels if I would provide palliative care for* [the resident]*, she would have more confidence in me now.* (WG2)  *This spring, we had a really nice* [conversation with the cards]*. And now during my parental leave she passed away, and then I have still thought about what a nice meeting we had before and that I got a little closer to her then.* (WG7)  *I think that in the future I will be more active also in that phase… some talk and think loud and others just… sort cards and almost do not talk for half an hour.* (I5)  *Maybe one should have played a larger role in some way … Well, you feel … less involved when there’s another person present and they’re talking a lot.* (WG5) | Evoking personal engagement |  |  |
| *I think this isn’t just a meeting,* [rather] *a process that must be complemented mutually. Sometimes you* [start] *but you don’t have time* … *and you have to end the conversation … and say ‘Okay, now we have started talking about it and I will return.’* (WG2)  *At first,* [the resident said he] *had absolutely nothing to talk to his son about, but after we had done* [the DöBra card exercise] *he thought that ‘Well, this might actually be good to bring up’.* (I3)  RN1*: But it is only positive that the husband was present* [in the EOL conversation]. *And then, I think* … *maybe* [any future] *conversation will be much easier too, when we know where he stands*.  RN2: *That's true. … then it becomes much more natural, that you can follow up that conversation when she gets worse.* (WG5) | Opening for continued communication | **Perceived impact** | **Perceived impact of card use in EOL conversations** |
| Participant*: I felt that somewhere he had gained more understanding. I think it was thanks to the cards because he no longer talked about needing to send her somewhere else, not even when I raised the issue. …*  Interviewer*: How was the conversation with him?*  Participant*:* … [while using the cards] *he started to think out loud… that if he himself would become demented, one would hardly want anything unnecessary but would want to have peace and quiet and have things be as good as possible. …* [So] *we were in full agreement in this final period, that we should try to make it calm* [for her]. *… And I connected it to the cards, that we had had this shared moment. … He said he had not thought of such things at all before.* (I5) | Aligning care goals among stakeholders |  |  |
| *I think it’s very strange, considering that in elder care people die all the time, it’s a fact.* … *you kind of just pussyfoot around* [the topic]*. No one mentions anything, neither nurses nor doctors, no one.* (WG2)  *I’ve had a little difficulty starting the conversation because I myself am afraid of death. Maybe you're right… I'm really scared. I do not want to die. Maybe that's why, it's a feeling I pass on to others as well and don’t dare to asking* [about]*…* (WG2)  *Almost all of us were also rather sceptical before, if you think about the first meeting, and now we are sitting here as well and are quite positive.* (WG5)  *Talking about death is a habit, for me anyway* … *but then you talk about it when it is approaching. Now you* [ask] *someone to try a* [card game]*, and then it's like* [residents think] *… ‘why are they talking about this game about death?’ And then it's like trying to almost apologize for bringing up this subject* (WG3)  [the name DöBra* cards] *makes you take it less seriously and* [people] *may not understand the purpose because you just hear ‘game’, it becomes silly*. (WG3)  *The DöBra* cards … is a tool to elevate the existential in all of us humans. … it’s such a great name, but … like the purpose of this is to be able to die well, but if you present in that way, then… I ask but is there any good way to die?* (WG2)  *DöBra is a Swedish pun literally meaning dying well and figuratively meaning awesome | Attitudes to engaging in EOL conversations | **Personal factors** | **Factors facilitating or limiting use or usability of the DöBra cards in RCH practice** |
| *If you’re not afraid yourself* … *if you dare to like ask questions when they say something, when they say that they are afraid of death. ‘What makes you afraid?’ And at that point*, *you start a conversation and then you can start talking about this* (WG2)  *Being very sensitive is important* … [Asking yourself] *what’s happening right now? It's hard to be like ... a robot in conversation. You have to adapt to the situation, that’s an important prerequisite.* (WG6)  *I understood that for her it wasn’t at all important to use the cards. She simply wanted to talk* … *And I listen.* … *Mostly approving* [what was said]*.* … *No, you don’t talk much. You mainly bring up the questions.* (WG6) | Facilitation style |  |  |
| *I felt during the conversation that she couldn’t handle it all at once, so I divided* [the conversation]. … *We waited a few days and then I went back because, with her concentration, it didn’t work, she thought it was too long.* … *I read* [the cards] *until she understood what I meant*. *She’s all there* [cognitively]*, but it was hard* [for her] *to decide.* (WG4)  *We should help people, we shouldn’t expose them to stress and anxiety and failure*. (WG6)  *But when it comes to* [residents with] *dementia, they don’t remember this. They may not even remember that they’ve done this, but they may have the feeling for a long time, that something is making them anxious and weighs them down and that they don’t feel really well. So that's why I'm pretty careful there to start testing with them. Because there I can see a risk that it may not be as clear and wonderful as [other conversations with the cards]…* (I4) | Adapting to residents’ varying needs | **Contextual factors** |  |
| *At first, I tried to sit in the communal room, it* [was too] *big* … *but the last* [conversation] *I had in the office. It went much better. Then you can concentrate on each other.* (WG6)  [staff] can’t do anything besides be involved in the conversation [during the allotted time]... Because you absolutely don’t want to be interrupted … so one needs to be completely spared [from other duties] for like an hour (WG6)  *Body language is important,* [to show] *that you’re in no hurry. Because that's what I felt. I didn’t want to show that I* [might need] *to rush out*. (WG6)  RN: *We have a section in* [the medical record system] *called* “*Own Wishes”*. *And that's only about the last stage* [in life]. *There you could… summarize and write down what has emerged.*  Interviewer: *But that’s a system only* [registered] *nurses* [can access]?  RN: *Yes, the RNs’ records. Is there anywhere* [assistant nurses] *can write?*  CNA: *No, we have nothing like that.*  (I4) | Organizational prerequisites for EOL conversations |  |  |

Note: *RN* – registered nurse, *CNA* – certified nursing assistant
